# The Transiliac Approach: A Forgotten Alternative for Transforaminal Endoscopic Decompression at the L5-S1 Level in Patients With High Iliac Crests

**DOI:** 10.7759/cureus.100149

**Published:** 2025-12-26

**Authors:** Ajay Krishnan, Abhijith Anil, Shivanand C Mayi, Ravi Ranjan Rai, Mirant B Dave, Kishor Murkute, Mikeson Panthackel, Arjit Vashishtha, Preety Krishnan, Bharat R Dave

**Affiliations:** 1 Spine Surgery, Bhavnagar Institute of Medical Sciences, Bhavnagar, IND; 2 Spine Surgery, Stavya Spine Hospital and Research Institute, Ahmedabad, IND; 3 Orthopaedics/Spine Surgery, Pushpagiri Institute of Medical Sciences and Research Centre, Thiruvalla, IND; 4 Radiology, Stavya Spine Hospital and Research Institute, Ahmedabad, IND

**Keywords:** awake spine surgery, day care surgery, lumbar disc herniation, minimally invasive spine surgery, transforaminal endoscopy, transiliac approach

## Abstract

Background

Transforaminal endoscopic lumbar discectomy (TELD) is a useful approach to tackle ventral compressive pathology in the spine. Its utility at the L5-S1 level is limited by anatomical factors such as a high iliac crest, large L5 transverse processes, large facets, narrow foramen, and the sacral ala. Transiliac transforaminal endoscopic lumbar discectomy (TI-TELD) has been reported to help overcome these obstacles, but has been reported sparsely.

Methodology

This was a single-center retrospective review of patients who underwent TI-TELD at the last mobile level in view of a high iliac crest at our center between January 2009 and January 2021. Only patients with complete records and a minimum two-year post-procedure follow-up were included. Patient records were scrutinized to note the demographic, surgical procedural details, and patient-reported outcome measures in terms of Visual Analog Scale and Oswestry Disability Index (ODI) scores.

Results

In total, 93 patients (83 men and 10 women) with a mean age of 41.77 ± 9.34 years were operated on over 12 years. Intraoperatively, it took a mean of 11.69 ± 2.38 minutes to reach the foramen. The mean operative time was 76.9 ± 12.24 minutes. Overall, 89 patients were discharged home on the same day, with decompression confirmed on MRI. The mean preoperative ODI score was 79.8%, and this improved to an average of 5.94% at the two-year follow-up and was maintained at the latest average follow-up of 59.98 months. In total, 82 of the 93 patients reported that they were highly satisfied with the procedure and gave a score of 1 as the patient satisfaction index. Overall, 13 of 16 patients with preoperative neurological deficits recovered Medical Research Council motor power greater than Grade 3. On average, our patients returned to work 19.20 days after surgery. Minor complications included a non-articular facet fracture (n = 1), prodrome to seizure (n = 1), ecchymosis (n = 4), superficial wound necrosis (n = 1), and bending of the obturator (n = 1). One patient required on-table conversion to micro-lumbar decompression. Postoperative MRI revealed an inadequate decompression in two patients, both of whom required fusion surgery. Two patients had a recurrent disc. Fusion was performed for the patient who had a recurrence on the same side, while the other patient was treated by TI-TELD from the opposite side.

Conclusions

TI-TELD is an under-reported procedure and provides good outcomes sustained over a longer follow-up than previously reported. Although more invasive than traditional TELD, the inherent benefits and advantages of being able to perform the procedure under local anesthesia make it more amenable for ambulatory surgical centers.

## Introduction

Transforaminal endoscopic lumbar discectomy (TELD) is an ultra-minimally invasive spine surgery (MISS) that can be done in a day-care ambulatory surgical setup through a minimal incision. The amount of blood loss and pain postoperatively is negligible. The possibility of execution under local/regional anesthesia, even applicability to morbidly obese or patients with comorbidities, and quicker resumption of work make it an exceptional procedure [1,2]. TELD is a bypass surgery that approaches ventral degenerative pathology (lumbar disc herniation (LDH) or ventral stenosis), avoiding retraction of neural tissue, damage to paraspinal muscles, and minimizing bony resection compared to conventional posterior decompression [3]. Lens optics under saline irrigation provides visualization, making it a full endoscopic spine surgery (FESS). Moreover, even postoperatively to TELD, MRI shows no scarring in the epidural space. This lack of scarring has been corroborated by findings on revision microdiscectomy (MLD) in patients with recurrence of LDH [4]. The nominal invasiveness is also demonstrated by the biochemical analysis of markers of inflammation and hidden blood loss after TELD [5,6].

TELD has been shown to be safe with good outcomes in patients with recurrent discs, migrated discs, foraminal discs, and even calcified discs. It has been used to effectively treat spondylodiscitis and to perform annuloplasty for the treatment of discogenic lower back pain. Lumbar canal stenosis affecting the lateral recess and foramen can also be managed by TELD [7]. Multilevel pathology, cauda equina syndrome, and nerve root anomalies are only relative contraindications, and several studies have demonstrated positive outcomes in such cases with TELD [7]. The recurrence rate following TELD is also comparable to the gold-standard MLD [4]. Although the applications of TELD are expanding, there are limitations to its utility at the L5-S1 level. The restrictions with TELD at L5-S1 were the precursor to the development of interlaminar endoscopic lumbar discectomy (IELD) [8,9]. A high iliac crest, large facets causing foraminal narrowing, large L5 transverse processes, and the sacral ala can come in the way of the working channel [10,11]. To circumvent these difficulties without compromising on the advantages of TELD, multiple techniques of foraminoplasty evolved along with a few isolated reports of transiliac transforaminal endoscopic lumbar discectomy (TI-TELD) [11-13]. Despite the possibilities with TI-TELD, it has not found widespread clinical application due to the limited clinical experience and limitations of the procedure [12,14-19]. The main impediment to the uptake of TI-TELD is the unreported perceived steeper learning curve compared to IELD and the increased invasiveness due to the reaming of the iliac crest [4,7,8,10,11,13,14,19-22]. The purpose of our study is to report the intricacies of the TI-TELD technique and a retrospective outcome analysis of our operated patients with a high iliac crest to demonstrate the efficacy and utility of this approach under local anesthesia (LA), along with its disadvantages.

## Materials and methods

Study design and participants

This was a single-center retrospective review of patients who underwent TI-TELD at the last mobile level at our center between January 2009 and January 2021. Informed consent was obtained from all included skeletally mature patients, who were informed of the need for open surgery if optimal decompression could not be performed. This study was approved after obtaining approval from the Institutional Ethical Committee of Stavya Spine Hospital and Research Institute (approval number: ECR/52/Inst/GJ/2013/RR-19). The study was registered with the Clinical Trials Registry - India (registration number: CTRI/2025/04/086043). The study adhered to STROBE guidelines for retrospective studies.

All TI-TELD planned patients were included in the study. A high iliac crest was defined as the highest point of the iliac crest being above the L5 pedicle level on the lateral view. All patients were operated on by the senior author (AK). Only patients with complete records and a minimum of two-year post-procedure follow-up were included. Patient records were scrutinized to note the demographic details and outcome measures. Inclusions were patients who had intra-canalicular LDH with protrusion, extrusion, migrated disc, or sequestrations with any associated variations at the reported last mobile levels, L4-5 and L5-S1 disc levels. Patients with adequate trial of progressive conservative modalities were recruited. Patients who did not consent to the procedure under LA, those with definite segmental instability, cauda equina syndrome, and malignancy were excluded. MRI scans were analyzed for the location and size of the LDH using the Michigan State University (MSU) classification [23], and migrated disc herniations were classified using the modified Lee’s classification [24]. The Ferguson view radiographs of the lumbar spine were assessed for the iliolumbar angle, while the lateral radiographs were assessed for the measurement of iliac crest height [25].

Intraoperative records were analyzed to identify the time taken for docking at Kambin’s triangle as well as the total procedure time. The number of fluoroscopic images acquired per procedure was also noted. The need for additional manoeuvres, such as foraminoplasty and alar reaming, to achieve adequate access to compressive discal pathology was noted. Pre- and post-procedure patient-reported outcome measures (PROMs) in the form of Visual Analog Scale (VAS) score for pain (back and dominant leg) and the Oswestry Disability Index (ODI) were noted. Intra- and postoperative complications, if any, were also noted. Muscle power was graded using the Medical Research Council (MRC) grade [26]. Power less than Grade 3 was considered significant, and recovery of power to more than MRC Grade 3 was considered objective recovery. The number of patients with objective motor power recovery was divided by the number of patients with significant preoperative motor weakness, and the result was multiplied by 100 to express the motor recovery rate. Postoperative MRI obtained within three hours of surgery was also reviewed by an independent blinded radiologist to assess the adequacy of decompression. The same radiologist assessed all MRI scans. Duration of hospital stay (in days) as well as the number of days taken to return to basic job or household work were noted. Patients were asked to rate their satisfaction with the procedure on a scale from 1 to 3, with 1 being fully satisfied and 3 being completely dissatisfied [27].

Surgical technique

**Figure 1:**
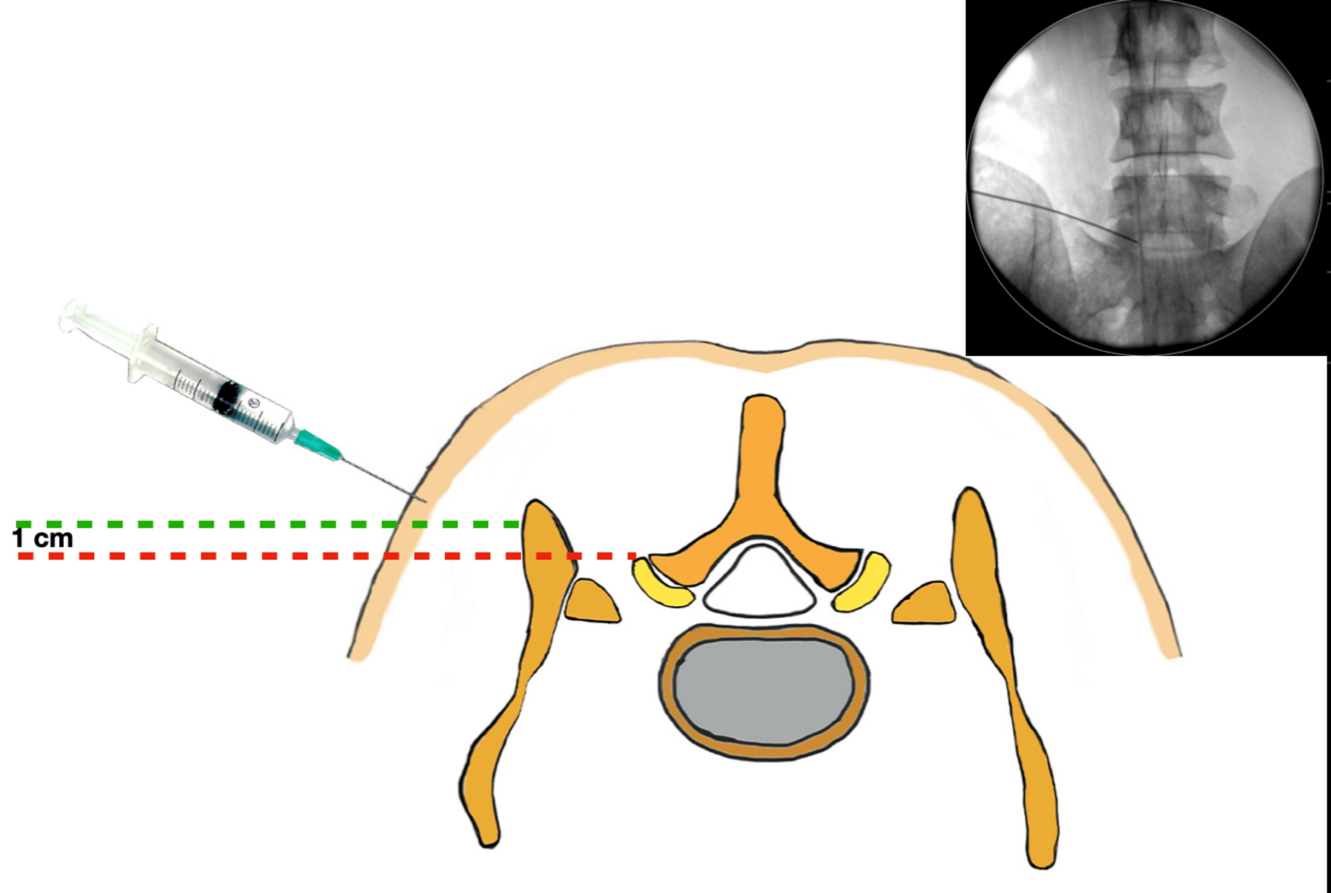
Illustration showing the intended needle skin entry site. Clinically, this point lies rostral to the posterior-superior iliac spine. The entry point on the iliac crest (green dotted line) was located within 1 cm of the posterior margin of the facet joint complex (red dotted line). The trajectory of the finally placed needle through the iliac window clears the alar inclination in the anteroposterior view (inset: anteroposterior fluoroscopy).

**Figure 2:**
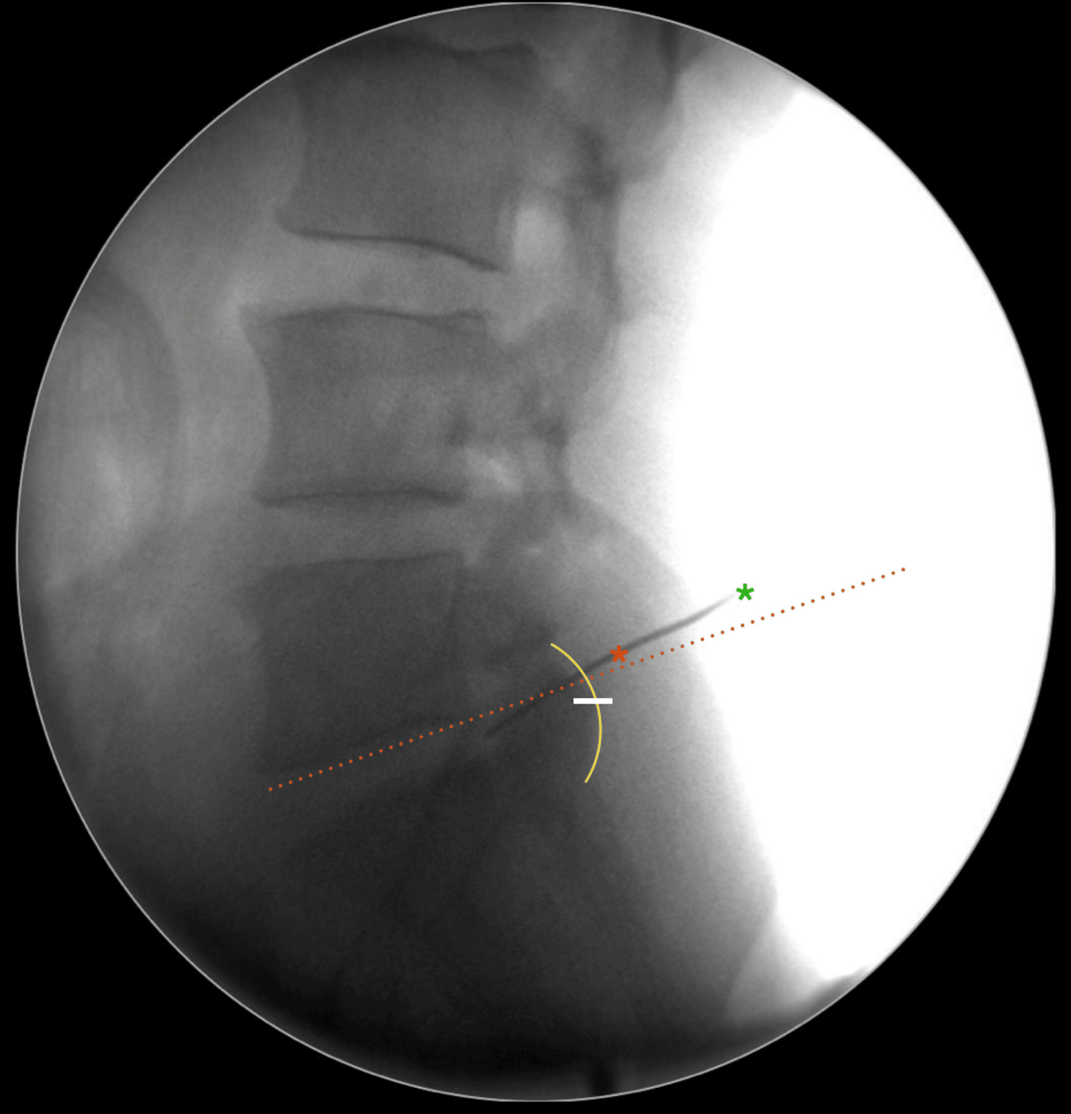
Fluoroscopic view after needle insertion. An imaginary line was drawn on the lower endplate of the L5 vertebra, as shown in lateral fluoroscopy (red broken line). The skin entry was around the projected region (green asterix). The intended needle entry point on the iliac crest was located within 1 cm of the posterior facet margin (yellow curved line) and named as “Krishnan’s point (K -point)” (red asterix). This hole was also above half (white marked line), with respect to the facet complex.

Patients were positioned prone on a radiolucent table with bolsters. Intramuscular diclofenac (75 mg) and midazolam (0.05 mg/kg) were administered half an hour before surgery. An intravenous dose of 1 µg/kg of fentanyl bolus was administered immediately before surgery. This was followed by additional doses as needed. A uni-portal TELD FESS approach was used from the side of maximum symptoms. Under fluoroscopic guidance, an end-on view of the L5 S1 disc with a squared L5 and S1 vertebra was obtained if possible (Ferguson view). The skin entry taken was around a projected region, as shown in lateral and anteroposterior C-arm fluoroscopy and illustration (Figures 1, 2).

**Figure 3:**
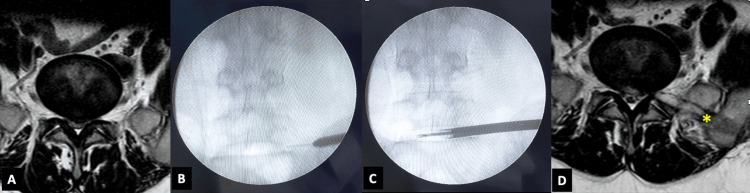
Needle insertion, access, and foraminoplasty in transIliac transforaminal endoscopic lumbar discectomy. (A) MRI of a 53-year-old male showing L5-S1 disc prolapse (Michigan State University Classification 1A), central disc prolapse. (B) The patient was operated on using transiliac transforaminal endoscopic discectomy. Iliac reaming is shown to 9 mm. Alar reaming was also needed. (C) The working cannula and endoscope are placed near the horizontal, para-centrally, with the reach of straight instruments, going to the opposite side. (D)  Immediate postoperative MRI shows adequate decompression. The co-planner iliac and alar reaming track is visible (yellow asterisk). The patient experienced significant symptom relief.

An imaginary line was drawn on the lower endplate of the L5 vertebra. It should be noted that this line clears the alar inclination in the anteroposterior view. If not, alar reaming was needed. The intended needle entry site was marked at 12 to 16 cm from the midline, depending on the build of the patient, and was infiltrated with LA of 1% lidocaine plus 0.25% bupivacaine in a 1:1 ratio. Then, through a 10 mm incision, a 16-G needle was advanced toward the Kambin’s triangle under fluoroscopic guidance until the iliac crest was encountered. In our regular practice, we found that the entrance point on the iliac crest was located within 1 cm of the posterior facet margin and named it the “Krishnan’s point (K-point).” This point can go more ventral or dorsal, making the need for foraminoplasty less or more, respectively. This point is also better within the rostral half of the facet complex to avoid alar reaming. The periosteum of the outer table was infiltrated with LA, following which a Jamshedi needle was placed to cross in a planned trajectory across the iliac crest, and again, LA was administered. Tom-shidi or alike reamers on the replaced guide pin were used to over-ream the iliac crest up to 9 mm. The sacral ala may have to be reamed for access. Then, over the guide pin, a 16-G needle was re-inserted with further infiltration around the facet into the epidural space and across the annulus. Then, over it, a tapered dilating obturator, a beveled working cannula, and then through that, the endoscope was introduced. Any of the system inventory as needed was used (Carl Storz-Gore system-Germany/Maxmore system, Germany; or Richard Wolf System, Germany). The surgery involved a FESS uniportal working channel with continuous irrigation, maintaining a clear field through maintained fluid pressure at the working area and washout of decompression debris. Irrigation was done with 0.9% saline using gravity and free drainage, with an intermittent manual pump being used as needed [28]. LDH excision was done along with the removal of compressive ventral tissues. The techniques employed depended upon the pathology. They were standard basic techniques of outside in (OI) or inside out (IO) with modifications as needed, such as alar reaming, additional endoscopic burred foraminoplasty, and calcified disc excision (Figure 3).

**Figure 4:**
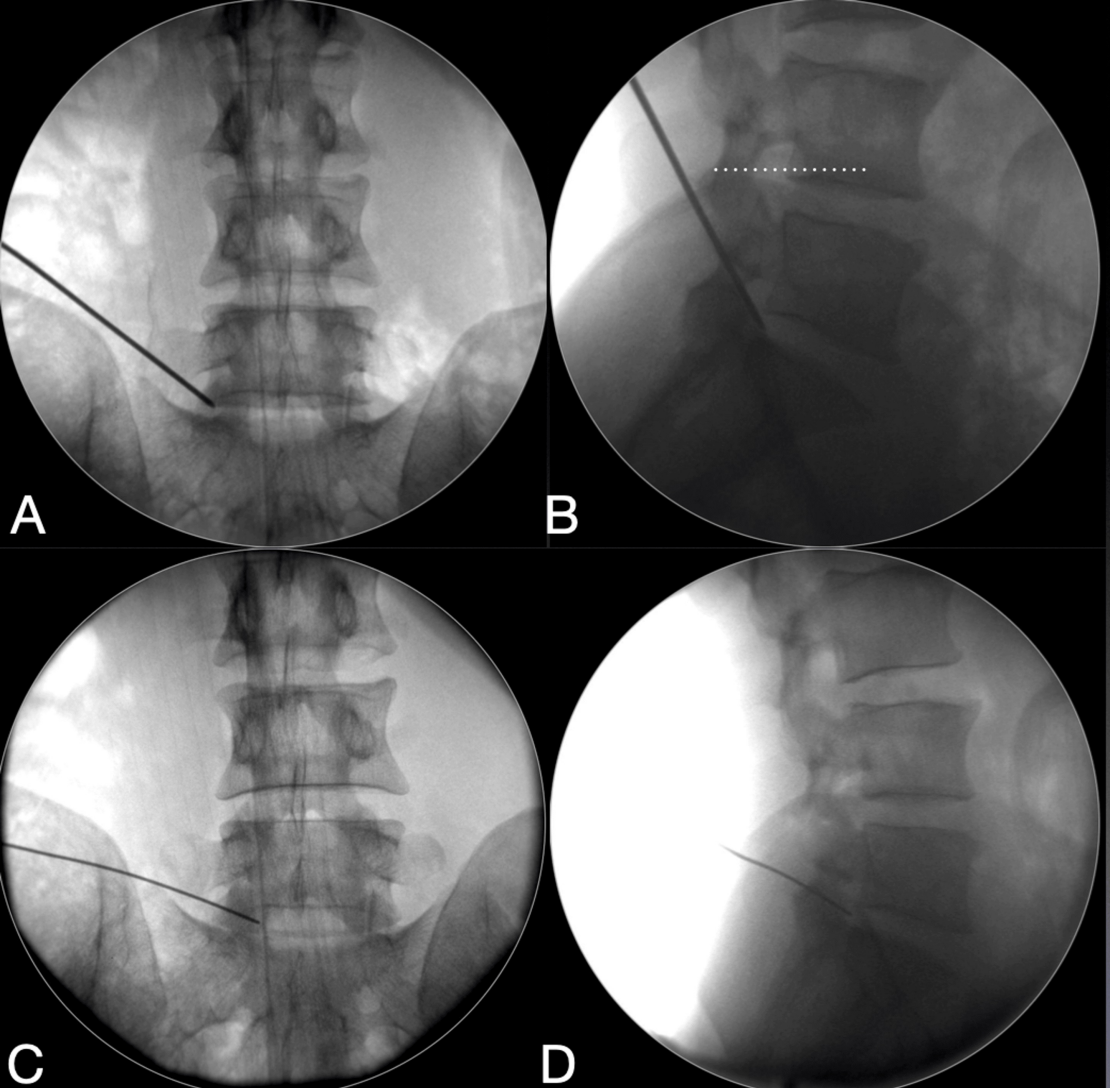
Difference in access with suprailiac and transiliac approaches in patients with a high iliac crest. A case of very high iliac crest (dotted white lines). Here, the first needle position obtained (A,B) on the planned supra-iliac attempt shows the oblique trajectory, with the tip of the needle at the non-ideal lateral pedicular line (A) and posterior discal level (B). For this index case, the endoscope could not reach optimally at the epidural medial location without substantial foraminoplasty. This trajectory may not allow reaching the central epidural area and impede complete verification signs of the endpoints of decompression. Moreover, endplate damage is likely to occur. Hence, in this index case, the approach was converted to the transiliac approach, as the case was a central big lumbar disc herniation (LDH). In the same case, transiliac needle position (C, D) after iliac crest tunnel access allowed medial pedicular position and coaxial approach, along the plane of the L5-S1. This is especially needed for high canal compromising big central LDH, hard calcified disc, up-migrating LDH, or across the side opposite ventral decompression.

**Figure 5:**
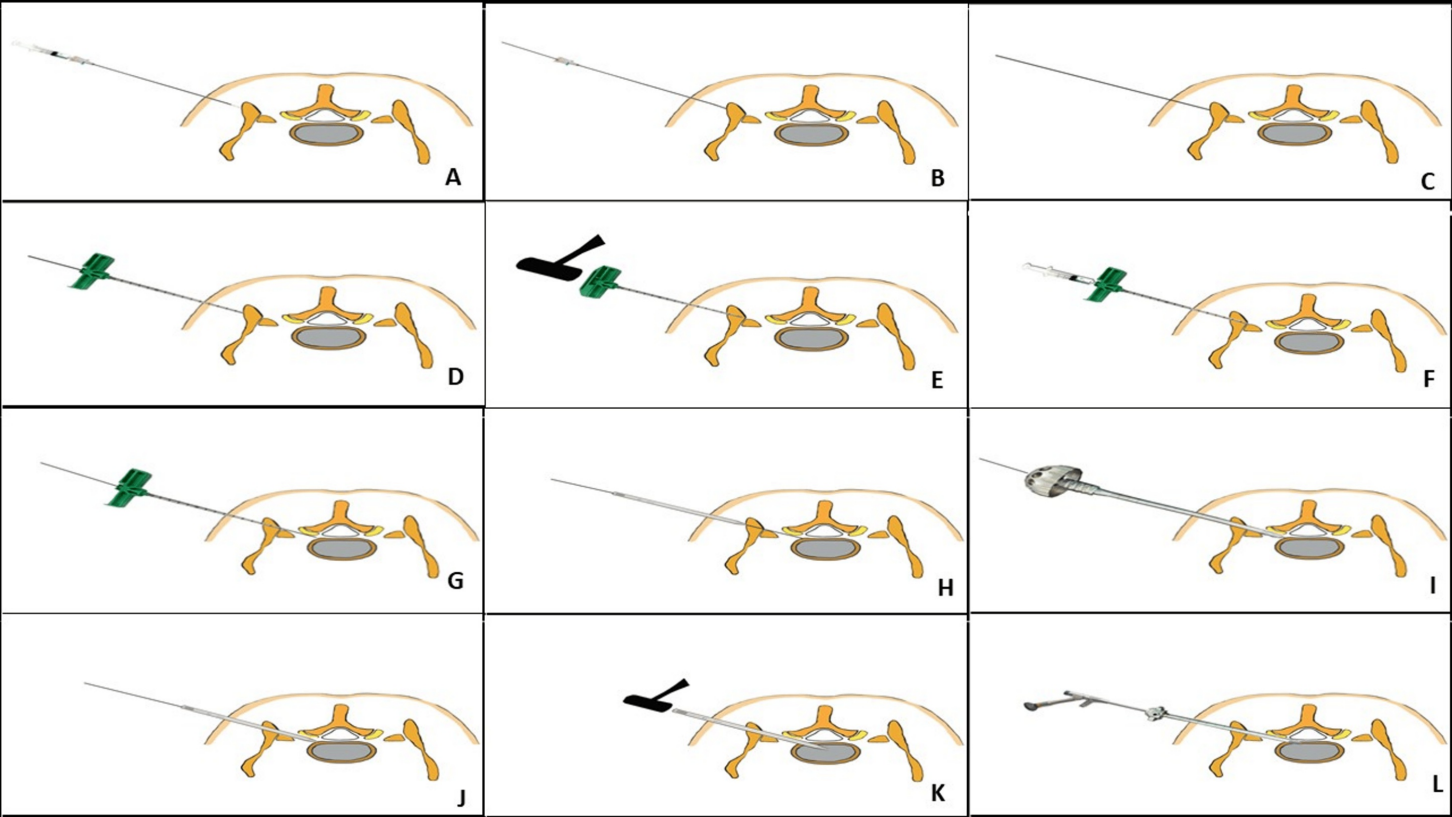
Technique of transiliac transforaminal endoscopic lumbar discectomy. (A) An 18-G needle inserted through a 10 mm incision and advanced to reach the optimal iliac crest site. (B) After administering LA, the needle stylet is withdrawn and the guide pin is railroaded. (C) The needle is withdrawn holding the guide pin in place. (D) A Jamshedi cannula (G) is introduced on the guidewire. (E) The guidewire is withdrawn. Jamshedi stylet is locked on the cannula T handle. Jamshedi needle is hammered across the crest to penetrate the internal side cortical bone. (F) LA is progressively infiltrated on the inner periosteum of the iliac crest and the area around the alar region. (G) The guide pin is introduced through the cannula after stylet withdrawal. (H) Obturator is put on the guide pin for tissue dilatation until the iliac crest and then withdrawn. (I) Sequential Tomshidi reamers are used up to 9 mm. The reamers used to ream the iliac crest channel can be advanced to ream the sacral ala and S1 superior articular facet if required. (J) Obturator is introduced over the guide pin. Fine adjustments of the trajectory can be done due to the over-reamed 9 mm bone tunnel to place the obturator tip at the medial pedicular line. (K) The guidewire is withdrawn and LA is administered on the surface of annulus and intradiscal. The obturator is hammered into the disc annulus. (L) The tapered working sheath is pushed over the obturator. Endoscope assembly is connected to initiate an inside-out approach. After sub-annular decompression, annulus is released to place the working sheath and endoscope more dorsally. Complete endpoint of decompression (EPD) is achieved in due course. In case of more medialization, horizontalization of the endoscope is needed for decompression of the opposite ventral side, and endoscopic visualized burred foraminoplasty is performed. This is needed when articulated/malleable hand instruments are not enough to visualize the EPD in soft disc prolapse and in hard calcified disc or when opposite side ventral decompression is needed.

Whenever there was a difficulty in a planned supra-iliac conventional approach to TELD at the last mobile level, a per-operative conversion to TI-TELD was performed (Figure 4). Decompression was ascertained to be adequate as per the objective assessments of endpoints of decompression, as described previously by the author [29]. Figure 5 demonstrates the entire technique in brief.

Statistical analysis

Descriptive statistics (mean ± standard deviation (minimum-maximum)) for all applicable variables were used to analyze the collected data. A paired t-test was applied to assess the significance of changes in PROMs post-surgery after confirming the normal distribution of data using the Kolmogorov-Smirnov test. A p-value below 0.05 was considered statistically significant. Lost to follow-up (LOF) patients after two years were still included in the data analysis. Failed and recurrent cases were excluded from outcome analysis except for mention in complications and for demography.

## Results

In total, 93 patients (83 men and 10 women) with a mean age of 41.77 ± 9.34 (range = 19-63 years) years were operated over 12 years. Overall, 73 patients had unilateral symptoms, while 20 had bilateral lower limb radicular pain. Further, 28 patients had up- or down-migrated disc herniations (zones 1 and 2). Six patients had cartilaginous and/or bony peripheral ring apophyseal fractures (PRAFs). Eight patients had calcified discs that caused ventral stenosis. Moreover, 16 patients had preoperative neurological deficits (MRC Grade <3) that manifested as weakness in plantar flexion. Eight patients were originally planned for conventional suprailiac TELD. However, per-operatively, they were converted to TI-TELD because of the inferred non-possibility of suprailiac approach while operating. Demographic variables are listed in Table 1.

**Table 1:** Descriptive demographic variables of the study participants. MRC = Medical Research Council; MSU = Michigan State University Classification; BMI = body mass index; PRAF = posterior ring apophyseal fracture/endplate cartilage fracture; LDH = lumbar disc herniation

Patient variable	Value
Age	41.77 ± 9.34 years (range = 19–63 years)
Sex	
Male	83
Female	10
BMI	28.32 kg/m^2 ^(±3.97)
Levels and gradings of iliac crest height	
Last mobile disc level	
L4-L5	9
L5-S1	84
Song et al. grade (above the L5 pedicle, n = 93)	
Grade II	89
Grade III	4
Iliolumbar angle (degrees)	47.10 ± 3.14 (36–57)
Iliac crest height (mm)	42.08 ± 2.12 (34–53)
Side of symptoms (number of patients)	
Unilateral	73
Bilateral	20
Conservative history (weeks)	11.45 ± 0.71 (2–28)
History of previous spine surgery	2
Location of LDH	
Central	45
Paracentral	48
MSU classification	
2A	2
2AB	23
3A	22
3AB	25
PRAF fragments	6
Calcified disc ventral stenosis	8
Migrated lumbar disc herniation	
Up-migrated	4
Down-migrated	23
Bi-directional migration	1
Magnitude of LDH migration, Lee’s grade	
Low migrated	20
High migrated	8
Neurological deficit (MRC Grade <3)	16
Comorbidities	
Hypertension	16
Diabetes mellitus	5
Hypothyroid	4
Bronchial asthma	2

Intraoperatively, it took a mean of 11.69 ± 2.38 minutes after marking to make the transiliac tunnel and to reach the foramen. The mean operative time was 76.9 minutes. On average, each procedure required 20.35 shots on fluoroscopy. Additional maneuvers of alar reaming (n = 4), burred/reamed foraminoplasty (n = 25), and calcified ventral stenosis decompression (n = 8) were performed when needed. The surgical variables and postoperative outcomes are tabulated in Table 2.

**Table 2:** Surgical and postoperative findings of the study population. For these patients, 24 months of follow-up data were available, which is included in the analysis. TI-TELD = transiliac transforaminal endoscopic discectomy

Surgical/Outcome-related variable	Value
Planned surgical approach	
Preplanned TI-TELD	85
Converted to transiliac per-operatively from the preplanned supra-iliac approach of TELD	8
Transiliac time taken to dock at the foramen (minutes)	11.70 ± 4.95 (5–21)
Need for additional maneuvers	
Alar reaming	14
Reamed Tomshidi foraminoplasty	4
Burred endoscopic foraminoplasty	21
Calcified ventral stenosis decompression	8
Mean TELD operative time (minutes)	76.9 ± 7.07 (46–119)
Number of fluoroscopic exposures	20.35 ± 2.12 (12–35 )
Complications	
Non-articular facet fracture	1
Prodrome to seizure	1
Unexpected damage to instruments	1
Incision site ecchymosis	4
Superficial wound necrosis	1
Inadequate decompression	2
Recurrent disc	2
Hospital stay (days)	1.04 ± 0 (1–2)
Return to work (days)	19.20 ± 2.12 (14–26)
Follow-up (months)	59.98 ± 34.23 (24–131); 7 patients were lost to final follow-up
Patient satisfaction index	1.13 ± 0 (1–2)

**Figure 6:**
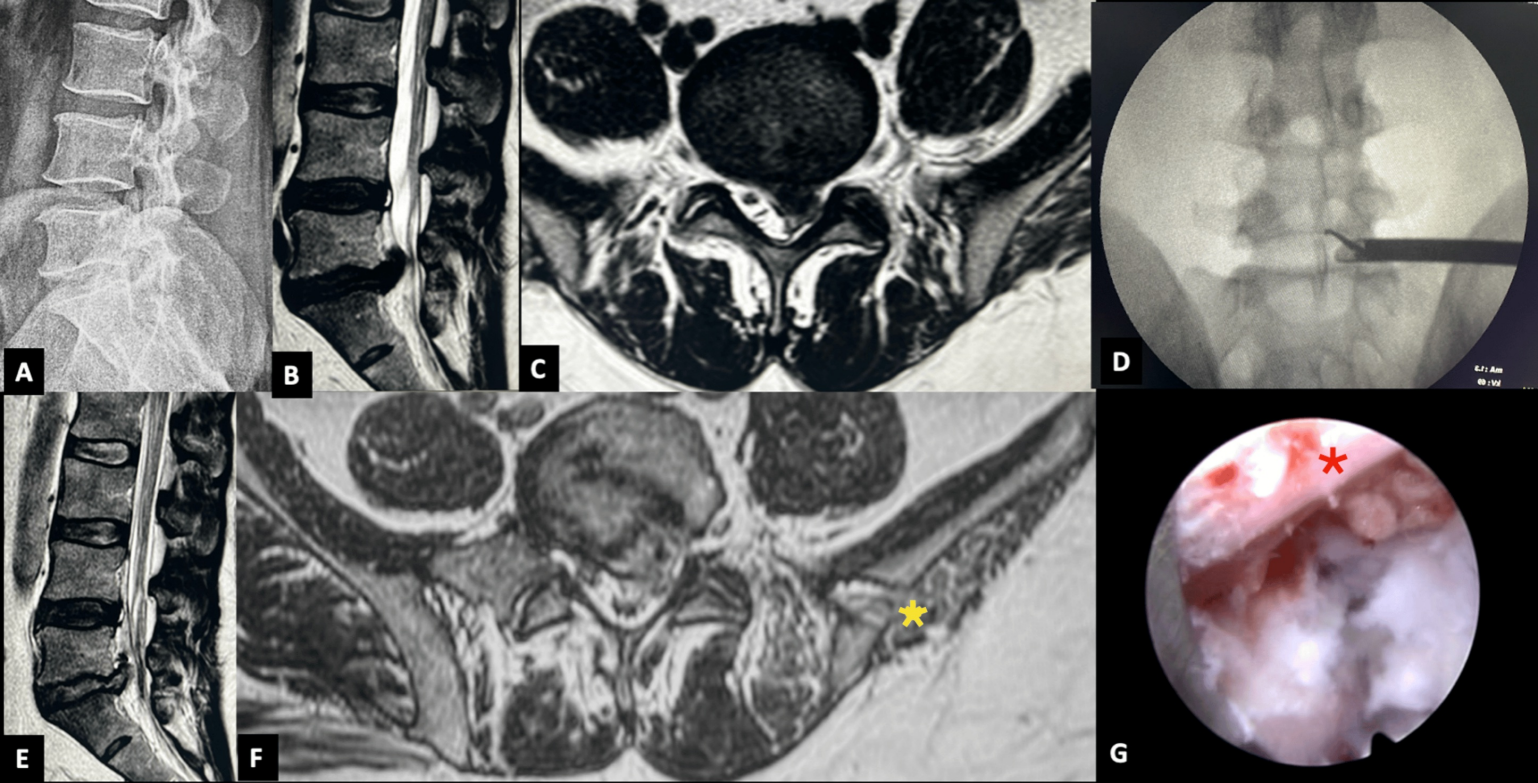
Transiliac transforaminal endoscopic lumbar discectomy in a patient with up-migrated lumbar disc herniation. (A) Lateral radiograph of a 41-year-old male with a high iliac crest. (B, C) MRI showing an up-migrated big disc fragment compressing the central dura and the left S1 root. (D) Per-operative C-arm image of the transiliac approach shows the horizontally positioned endoscope assembly and the hook used to tease the up-migrating fragment. (E) Postoperative MRI showing adequate decompression. (F) Oblique MRI showing the transiliac channel (yellow asterisk) and adequate decompression. (G) Adequacy of decompression with the decompressed pulsatile traversing nerve root (red asterisk).

The mean preoperative ODI score was 79.8%. This improved to an average of 17.7 at the three-month follow-up, 10.63 at the sixth-month follow-up, and 5.94 at the two-year follow-up. This improvement was noted to be statistically significant (Table 3). This was also greater than the minimal clinically important difference (MCID) of 30 percentage points reported for the ODI score and 3 points for the VAS score (Tables 3-5, Figure 6) [30].

**Table 3:** Functional outcomes of the transiliac transforaminal endoscopic discectomy (ODI scores) ODI = Oswestry Disability Index

Time point of measurement	Mean	Standard deviation	Range
Preoperative	79.8	13.12	63.2–95.6
3 months	17.7	4.64	10–30
6 months	10.63	3.79	2.2–22
24 months	5.94	3.07	2.2–10
Latest follow-up	6.38	3.61	2.2–18

**Table 4:** Functional outcomes of the transiliac transforaminal endoscopic discectomy (VAS for back pain). VAS = Visual Analog Scale

Time point for measurement	Mean	Standard deviation	Range
Preoperative	6.34	2.52	0–10
Postoperative	1.68	0.54	0–3

**Table 5:** Functional outcomes of the transiliac transforaminal endoscopic discectomy (VAS for leg pain). VAS = Visual Analog Scale

Time point of measurement	Mean	Standard deviation	Range
Preoperative	9.24	0.8	2–10
Postoperative	0.80	0.82	0–3

**Figure 7:**
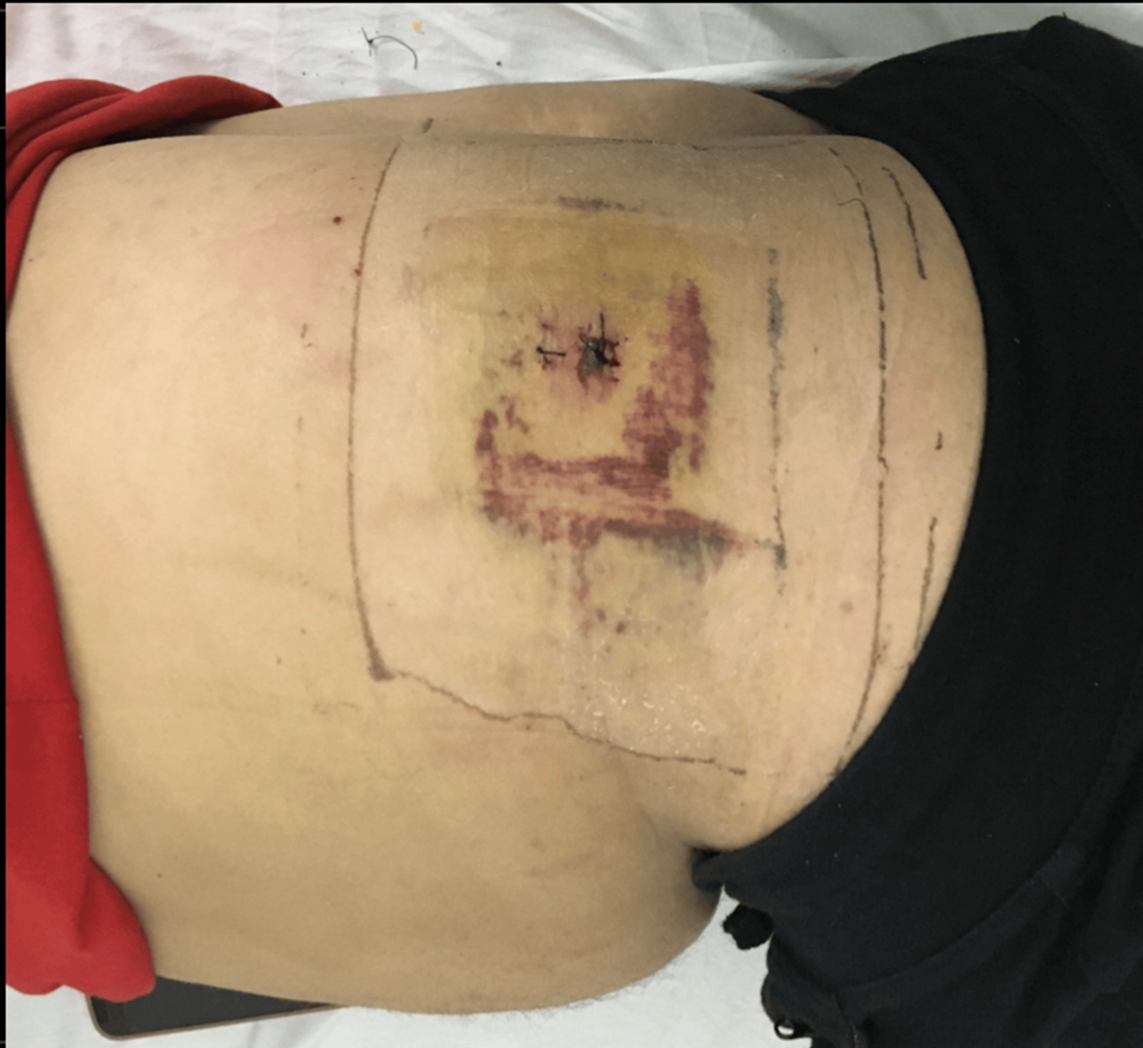
Ecchymoses at the incision site. Image of the first dressing on third day of the surgery, showing ecchymosis around the entry point of the transiliac approach. No medications or treatment alterations were needed, and there was no eventual effect on immediate or final excellent outcome.

**Figure 8:**
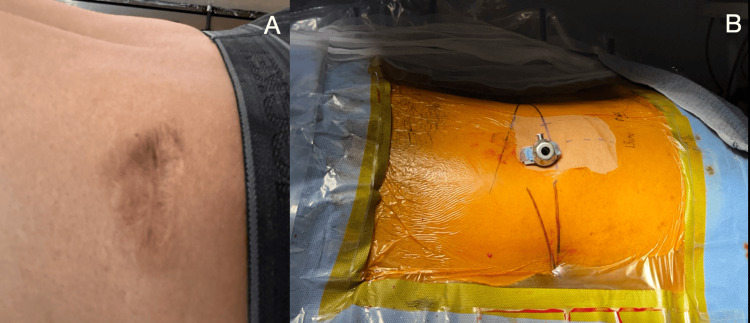
Superficial wound necrosis at the incision site leading to a puckered scar formation. (A) A case of transiliac approach endoscopy with superficial necrosis which led to secondarily healed puckered scar. (B) These scars can occur during a long surgery, in which the working sheath is pushed to its hilt at entry points 14-16 cm in obese/overweight patients.

Overall, 13 of 16 patients with preoperative neurological deficit recovered MRC motor power greater than Grade 3. On average, our patients returned to work 19.20 days after surgery. Further, 82 of the 93 patients reported that they were highly satisfied with the procedure and gave a score of 1, while seven patients were satisfied with the procedure and chose a score of 2. The remaining four patients with a score of 3 required conversion to a fusion or MLD procedure. One patient developed a non-articular facet fracture following foraminoplasty, which was noted intraoperatively. The fracture did not require any further management. One case failed execution and required conversion to MLD immediately. One patient developed symptoms of raised epidural pressure intraoperatively, manifesting as prodrome to seizure, but the procedure could be completed by TI-TELD after raising the head and halting for 15 minutes. Four patients developed ecchymosis at the incision site (Figure 7). One patient developed superficial wound necrosis that healed by secondary intention, leading to the development of a puckered scar (Figure 8). Overall, 89 patients were discharged home on the same day, with decompression confirmed on MRI and satisfactory immediate and long-term outcomes.

**Figure 9:**
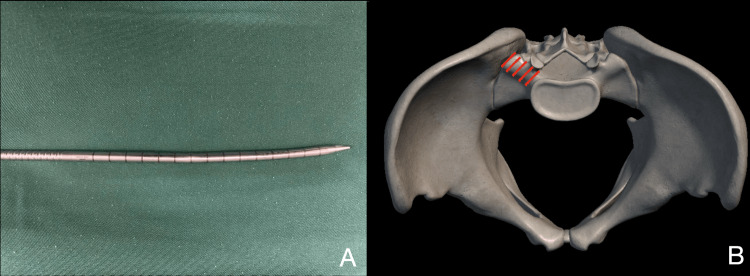
Damage to instruments after transiliac transforaminal endoscopic lumbar discectomy. (A) Bend of the obturator in the transiliac approach. (B) The shaded portion of the sacral ala that may be thick cortical bone. It should be avoided in the approach or reamed to avoid any damage to the instrument.

Postoperative MRI revealed an inadequate decompression in two patients. One of these had conjoined roots and complained of persistent dysesthesia post-procedure. The other patient had an associated calcified disc causing ventral stenosis. Both of these patients required fusion surgery. Two patients had a recurrent disc after TI-TELD, one on the same side and one on the opposite side, within three months. Fusion was performed for the patient who had a recurrence on the same side, while the other patient was treated by TI-TELD from the opposite side. Other complications were damage to the instruments used, leading to bending of the obturator (Figure 9).

## Discussion

MISS surgeries and further FESS have emerged as a bridge on the scale from conservative treatment to traditional open surgery. IELD approaches have evolved and have been reported robustly in the literature, citing the intricacies and invasiveness of TELD at the L5-S1 level [8]. At the same time, foraminoplasty has emerged as a saviour to enhance the reach of TELD at L5-S1, retaining the core advantages of the transforaminal approach [13,14]. To avoid failure in accessing the L5-S1 level by TELD, a classification has been described to help decide between suprailiac TELD or the TI-TELD by Patgaonkar et al. [15].

The iliac crest can hamper a coplanar access to the disc, and taking a more medial and superior entry can help avoid the iliac crest to obtain access to the disc using an oblique trajectory. However, this trajectory precludes a complete discectomy, as the facet, along with the iliac crest, prevents the scope from being angled ventrally and caudally to clear the epidural space fully. Foraminoplasty is the only solution [10]. Although foraminoplasty works, many reports of blind reamed foraminoplasty have noted dysesthesia of the exiting root [14,20].

Osman et al. (1997) first published a report on the feasibility of using a TI-TELD on the L5-S1 disc for endoscopic discectomy in a cadaveric study [13]. They noted that the track traversed the gluteus medius and minimus muscles. The track was reported to be an average of 1.8 cm (range = 1.5 to 2.3 cm) from the lumbosacral trunk. The superior gluteal neurovascular bundle was, on average, 4.37 cm from the track. The track did not injure the ilio-lumbar ligament in any of the cadavers [13]. Choi et al. classified patients based on the height of the iliac crest into six grades, with the highest grade being a patient with an iliac crest height higher than the L5 pedicle, in addition to the iliac height and ilio-lumbar angle [31]. There is no universally accepted definition of a high iliac crest. Song et al. used a higher grade of classification with reference to the L4 pedicle [32]. All of our patients (n = 93) managed by TI-TELD had disease at the last mobile level with a high iliac crest greater than Song Grade 2 and corresponding to Choi Grade 6, indicating how our approach overcomes the impedance posed by the high iliac crest. Objectively, the average height measurements over the S1 upper endplate (42.08 mm) and ilio-lumbar angle (47.1 degrees) of our study population are higher than those reported by Choi [31]. A projected coplanar access to the L5-S1 disc overlaps the ilium more commonly in male patients [33]. This was noted in our study population as well, which was predominantly male (n = 83, 89.25%).

The first clinical report of TI-TELD on patients was published as a technical report by Choi et al. (2009). They performed TI-TELD in two patients with a foraminal disc at the L5-S1 disc in patients with high iliac crests [12]. A few more studies were published following this study, and the improvement in PROMs was remarkable in all reports [12,14,15,17,18]. An average of 5-20 minutes was needed for preparation of the transiliac tunnel in their patients [12,17] compared to 11.7 minutes in our series. Our much larger cohort of patients showed similar results, which were sustained over a two-year follow-up and the latest follow-up average of 59.98 months (Figure 9). The follow-up in the literature published so far has no more than 12 months of follow-up with small patient cohorts (n = 2, 15, 10, 19, 44, respectively) [12,14,15,17,18].

No patients had major complications in the previous TI-TELD literature. No major complications occurred in any case in our series either. One patient with failed execution was converted to MLD, and two patients with inadequate decompression were fused subsequently. All three cases were in the early learning experience. One patient with a recurrent disc was fused in due course. In another patient with recurrence from the opposite side, TI-TELD was performed. This again reinforces the patients’ satisfaction with the procedure.

**Figure 10:**
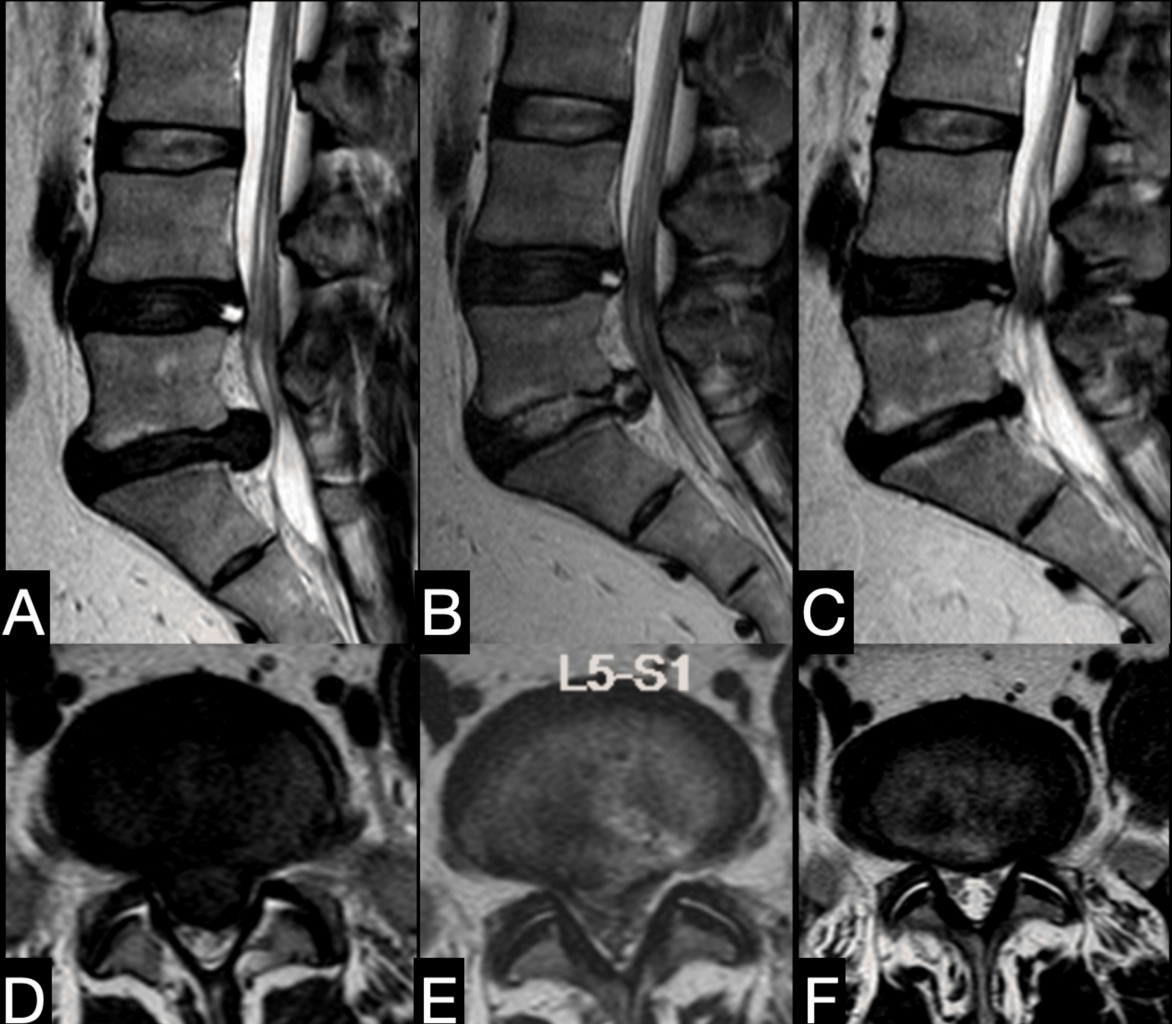
Large central disc operated by transiliac transforaminal endoscopic lumbar discectomy with adequate decompression demonstrable on postoperative MRI. (A, D) MRI of a 49-year-old male with a high iliac crest showing L5-S1 big disc prolapse, Michigan State University Classification 3AB, central disc prolapse with non-symptomatic L4-5 annular tear. The patient was operated on using transiliac transforaminal endoscopic discectomy. (B, E) Immediate postoperative MRI shows adequate decompression. The patient had significant relief of symptoms. (C, F) Nine-year follow-up MRI with maintained radiological and excellent clinical outcome at the L5-S1 and static L4-5 annular tear.

**Figure 11:**
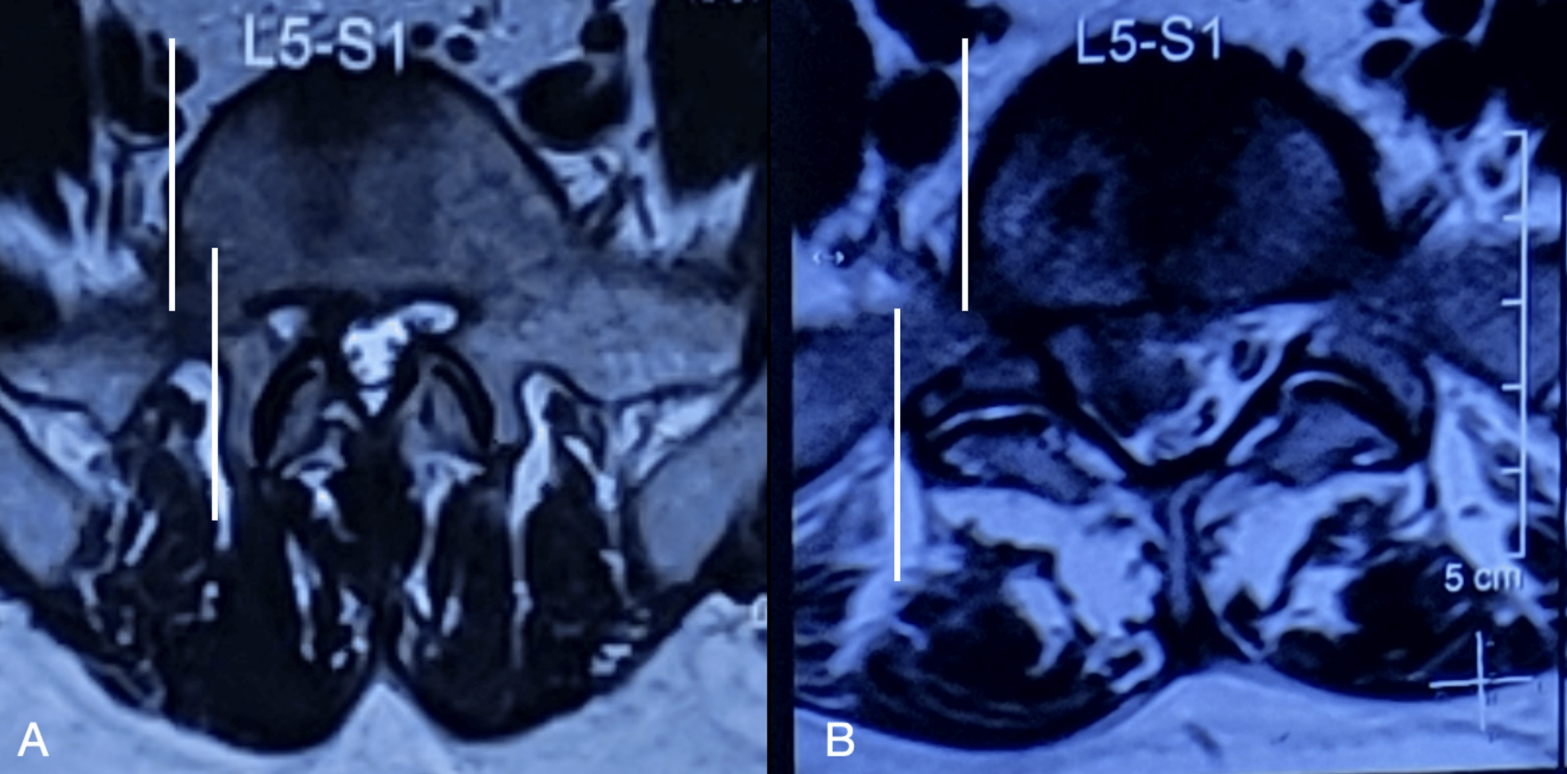
Identification of large facets for the purpose of planning transiliac transforaminal endoscopic lumbar discectomy. (A, B) MRI T2 axial section of two patients. Note the marked difference in orientation of facet (bulbous and coronal), its size (bigger), wider interpedicular distance, and projection of facet margin beyond the vertebral body margin (white demarking lines) in Panel B compared to Panel A. This makes it difficult to reach a disc fragment in Panel B. A flatter transiliac trajectory and/or a wider foraminoplasty will be needed in the second case.

The use of the TI-TELD will allow the surgeon to address central pathology and even the opposite lateral recess in view of the more direct angle of approach, as known and reported previously in complex pathologies and LDH [2,7,15]. The difficulties with the use of TI-TELD include the narrow range of movement of the working cannula and endoscope combo, the possibility of iliac fractures, superior gluteal artery injury, and gluteal epithelial nerve damage [31,34]. Another disadvantage of the use of TI-TELD is the increased exposure to ionizing radiation. We have noted the average time taken to cross the iliac crest (11.70 minutes) and the number of fluoroscopic exposures (n = 20.35) done, which are comparable with the reported literature [12,17,18]. Ahn et al., quantifying whole body radiation exposure per year, reported that 5,379 surgeries can be performed with a lead apron, whereas only 291 operations can be performed without a lead apron [35]. In the future, the use of navigation technology can limit the radiation exposure and operation time in endoscopy [7,35]. TELD is associated with a steep learning curve, and this learning curve is even higher at the L5-S1 level [36,37]. Potentially, this could mean a much steeper learning curve for performing TI-TELD. Many words are often used differently in common parlance of science, and one of them is the usual misconception that steep means difficult. Contrastingly, the steepness means an easier learning curve than a plateau learning curve [36,37]. Regarding unsuccessful TELD cases, a single-center experience (n = 10,228) reported that a non-ideal working channel position played an important role [22]. At the same time, multiple punctures to arrive at a correct position in L5-S1 is a big deterrent to TELD, adding to the learning curve. To increase the ease of introduction and reduce radiation exposure, multiple techniques have been reported, including cumbersome jigs, the use of navigation, and the lumboiliac triangle bull’s eye docking technique [38-40]. This challenge was also reflected in our data. Eight patients who were initially planned for a supra-iliac TELD were converted to a TI-TELD instead of a foraminoplasty. This was per-operatively changed as the best needle position obtained could not achieve an optimal trajectory (Figure 3). All of these eight patients had excellent outcomes, with a patient satisfaction index score of 1. This happened early in our experience, and now we have identified indications to primarily attempt TI-TELD, as when there is a large central disc, the facets are bigger and wider, along with high iliac crests (Figures 10, 11). Additionally, considerations in such cases are complex index pathology (hard calcified discs, soft upmigrated discs, and large central LDH with hard components who are bilaterally symptomatic).

The standout difference between TELD and IELD is the principle itself. Interlaminar access exposes neural tissue first and then addresses the disc. However, in TELD, we complete the work on the disc first, and only then, the neural structures come into view. This neural handling can make it difficult for patients to tolerate IELD under LA and increases the risk of neural injury [34]. Two randomized controlled trials have been performed comparing IELD and TELD at the L5-S1 level under LA to date, with some contradictory findings. Chen et al. reported in the TELD group a shorter operative time, postoperative bed rest time, and lower VAS scores for intraoperative back pain and leg pain than the IELD group. However, on postoperative follow-up, there were no significant differences in PROM except the surveys showing a significantly higher satisfaction rate in the TELD group. Moreover, six patients for intense procedural pain in the IELD group needed extra intravenous injections of sufentanil with an additional two cases of neuropathic pain after surgery [41]. Nie et al. reported a higher mean operation time in TELD, but no significant difference in PROM, postoperative bed rest time, hospitalization time, or complication rate [42].

In our series, all patients were managed as day care (1.04 days average hospital stay), and the average return to basic work or job was 19.20 days after surgery. The patients’ satisfaction index with our procedure was 1, highly satisfied, in 88.17% patients (n = 82). This also justifies the feasibility in LA. Table 6 provides an extensive literature review to emphasize the learning points.

**Table 6:** Summary of published literature on TI-TELD. TI-TELD = transiliac transforaminal endoscopic lumbar discectomy; VAS = Visual Analog Scale; ODI: Oswestry Disability Index; NR = not reported

Study (year)	Number of patients	Objective high iliac crest definition	Average time to dock at foramen (minutes)	Average fluoroscopic exposures	Average procedure time (minutes)	Length of hospital stay (days)	Outcome VAS/ODI (at final follow up)	Final average follow-up (months)	Technique/Remark
Choi et al. (2009) [12]	2	Iliac crest not objectively described	5 to 7 minutes	NR	NR	NR	NR	NR	First pioneering work as a technical note. Discography was done
Osman et al. (2014) [14]	15	Iliac crest not objectively described	NR	NR	NR	NR	VAS Improved to 3.6 for back pain and 2.9 for leg pain, and ODI improved to 16.7%	12	For decompression, sedation and local anesthesia are used. Chromodiscography was done. Guidewire was drilled through the iliac crest and to the posterolateral disc over a predetermined trajectory with a power drill. Thereafter, the core reamer was used. For fusion cases, general anesthesia was used. Some patients underwent fusion with expandable cage inserted. Neuromonitoring was used
Mahesha et al. (2016) [17]	10	Iliac crest not objectively described	20 minutes	NR	70	1 day	VAS improved to 1.6 for leg pain, and ODI improved to 6%	12	A K-wire/Beath pin was first positioned by drilling across the iliac crest and then reaching the disc. A Flower tip cannulated reamer was used from anterior cruciate knee arthroscopy reconstruction set
Bai et al. (2017) [18]	19	Choi et al. Grade I to VI	NR	17	86	5 days	VAS for leg pain improved to 1	12	The study had a comparative group of non-L5-S1 compared with the technique of TI TELD with no major outcome difference, including operative time and radiation measures. In the transiliac approach, a CT measurement criteria was used with an objective point of entry localized in lateral per-operative radiograph. The superior gluteal artery vascular malformation was checked by ultrasound before surgery. The iliac hole was made bigger to 10 mm, and then the needle was positioned to apt location of the disc
Patgaonkar et al. (2020) [15]	44	Own objective classification with respect to anteroposterior and lateral radiographic measurements and reference to L5 pedicle	NR	NR	NR	1 day	VAS for leg pain improved to 2.27, and ODI improved to 15.72%	6	The study describes the decision-making to choose supra-iliac or infrailiac approaches based on radiological parameters. Sequential reamers (Maxmore GmbH) were used for the iliac window. Foraminoplasty was invariably used in most of the cases
DeAssiss et al. (2024) [43]	2	Utilized the classification system described by Patgaonkar et al.	NR	NR	50	1 day	VAS for leg pain improved to 1 and for back pain to 2.5. The ODI score improved to 17%	24	A case series of 2 patients with a high iliac crest treated by the TI approach at the L5-S1 level. Both patients had good postoperative outcomes that were sustained over a two-year follow-up
Our series	93	Song et al. Grade II and III , equivalent to Choi et al. Grade VI	11.70 minutes	20.35	76.9	1 day	VAS improved to 1.68 for back pain and 0.80 for leg pain, and ODI improved to 6.17%	59.19	The largest series with a long-term outcome reported. All cases were of high iliac crest, high grade. Successful objective, decompression by the inside-out technique, could be achieved in all but two and one failed execution. A very limited number of complications and recurrence was noted. The step-by-step approach is discussed extensively

To make TI-TELD perpetuating and reproducible, the technique has been described methodologically with highlighted complications and pitfalls to ease learning points. However, the risks and benefits must be further correlated to other surgical approaches with the surgeon’s preferences in clinical practice.

In the last five years, there has been a renewed interest in the Transkambin endoscopic approach for fusion worldwide [44,45]. It is not surprising to find sporadic reports of transiliac interbody fusion as well [46]. Principally, if we want to take the full advantage of TELD with LA and evolve to advanced indications, rather than switching to an alternative approach of interlaminar endoscopy, despite the blockages of the anatomical structures, a foraminoplasty or TI-TELD is the way out. This would add value to the principles of enhanced recovery after surgery and awake spinal surgery [47].

The main limitation of this study is its retrospective design. Certain subgroups of patients within the 93 patients, such as those with migration, calcified discs, and those with PRAFs, were small. Hence, the statistical power to detect differences in outcomes among these patients is small, and further research is required. Individual anatomic-morphometric parameters differences, such as obesity, gender, height, iliac angle, facet shape, alar flare, global spinal alignment, facet projection of facet beyond the vertebral body margins, interpedicular distance, and transitional vertebra, would have been confounding factors, which were not considered, but may influence the approach and outcome. However, the present study aimed to report the technicality and clinical results of TI-TELD for the lumbo-sacral junction and not epidemiology, or to compare it with other methods. Other limitations relate to the potentially time-consuming and invasive approach to inventory logistics. Moreover, most of the inferences are based on the senior author’s experience of 15 years in TELD.

## Conclusions

TI-TELD can overcome the barrier of iliac crest and facet challenges and is an effective tool for surgeons who perform TELD. TI-TELD allows coplanar access to the disc and anterior epidural space without neural retraction. TI-TELD is safe and is associated with excellent patient outcomes that are sustained over long-term follow-up.

## References

[1] Hofstetter CP, Ahn Y, Choi G (2020). AOSpine consensus paper on nomenclature for working-channel endoscopic spinal procedures. Global Spine J.

[2] Gadjradj PS, Rubinstein SM, Peul WC (2022). Full endoscopic versus open discectomy for sciatica: randomised controlled non-inferiority trial. BMJ.

[3] Krishnan A, Murugan C, Panthackel M (2024). Transforaminal endoscopic ventral stenosis decompression in calcified lumbar disc herniation: a long term outcome in 79 patients. World Neurosurg.

[4] Ruetten S, Komp M, Godolias G (2005). An extreme lateral access for the surgery of lumbar disc herniations inside the spinal canal using the full-endoscopic uniportal transforaminal approach-technique and prospective results of 463 patients. Spine (Phila Pa 1976).

[5] Zhuang H, Li J, Guo S (2024). Hidden blood loss in three different endoscopic spinal procedures for lumbar disc herniation. Ann Med Surg (Lond).

[6] Barber SM, Nakhla J, Konakondla S, Fridley JS, Oyelese AA, Gokaslan ZL, Telfeian AE (2019). Outcomes of endoscopic discectomy compared with open microdiscectomy and tubular microdiscectomy for lumbar disc herniations: a meta-analysis. J Neurosurg Spine.

[7] Krishnan A, Kim HS, Raj A (2021). Expanded indications of full endoscopic spine sugery. J Minim Invasive Spine Surg Tech.

[8] Choi G, Lee SH, Raiturker PP, Lee S, Chae YS (2006). Percutaneous endoscopic interlaminar discectomy for intracanalicular disc herniations at L5-S1 using a rigid working channel endoscope. Neurosurgery.

[9] Ruetten S, Komp M, Merk H, Godolias G (2008). Full-endoscopic interlaminar and transforaminal lumbar discectomy versus conventional microsurgical technique: a prospective, randomized, controlled study. Spine (Phila Pa 1976).

[10] Tezuka F, Sakai T, Abe M (2017). Anatomical considerations of the iliac crest on percutaneous endoscopic discectomy using a transforaminal approach. Spine J.

[11] Lee SH, Kang HS, Choi G, Kong BJ, Ahn Y, Kim JS, Lee HY (2010). Foraminoplastic ventral epidural approach for removal of extruded herniated fragment at the L5-S1 level. Neurol Med Chir (Tokyo).

[12] Choi G, Kim JS, Lokhande P, Lee SH (2009). Percutaneous endoscopic lumbar discectomy by transiliac approach: a case report. Spine (Phila Pa 1976).

[13] Osman SG, Marsolais EB (1997). Endoscopic transiliac approach to L5-S1 disc and foramen. A cadaver study. Spine (Phila Pa 1976).

[14] Osman SG, Sherlekar S, Malik A, Winters C, Grewal PK, Narayanan M, Gemechu N (2014). Endoscopic trans-iliac approach to L5-S1 disc and foramen - a report on clinical experience. Int J Spine Surg.

[15] Patgaonkar P, Datar G, Agrawal U, Palanikumar C, Agrawal A, Goyal V, Patel V (2020). Suprailiac versus transiliac approach in transforaminal endoscopic discectomy at L5-S1: a new surgical classification of L5-iliac crest relationship and guidelines for approach. J Spine Surg.

[16] Chen KT, Wei ST, Tseng C, Ou SW, Sun LW, Chen CM (2020). Transforaminal endoscopic lumbar discectomy for L5-S1 disc herniation with high iliac crest: technical note and preliminary series. Neurospine.

[17] Mahesha K (2018). Endoscopic transiliac approach to L5-S1 disc and foramen, technique and results. J Orthop Allied Sci.

[18] Bai J, Zhang W, Wang Y (2017). Application of transiliac approach to intervertebral endoscopic discectomy in L5/S1 intervertebral disc herniation. Eur J Med Res.

[19] Chae KH, Ju CI, Lee SM, Kim BW, Kim SY, Kim HS (2009). Strategies for noncontained lumbar disc herniation by an endoscopic approach : transforaminal suprapedicular approach, semi-rigid flexible curved probe, and 3-dimensional reconstruction CT with discogram. J Korean Neurosurg Soc.

[20] Yeung AT, Tsou PM (2002). Posterolateral endoscopic excision for lumbar disc herniation: surgical technique, outcome, and complications in 307 consecutive cases. Spine (Phila Pa 1976).

[21] Gibson JN, Cowie JG, Iprenburg M (2012). Transforaminal endoscopic spinal surgery: the future 'gold standard' for discectomy? - A review. Surgeon.

[22] Choi KC, Lee JH, Kim JS, Sabal LA, Lee S, Kim H, Lee SH (2015). Unsuccessful percutaneous endoscopic lumbar discectomy: a single-center experience of 10,228 cases. Neurosurgery.

[23] Mysliwiec LW, Cholewicki J, Winkelpleck MD, Eis GP (2010). MSU classification for herniated lumbar discs on MRI: toward developing objective criteria for surgical selection. Eur Spine J.

[24] Lee S, Kim SK, Lee SH, Kim WJ, Choi WC, Choi G, Shin SW (2007). Percutaneous endoscopic lumbar discectomy for migrated disc herniation: classification of disc migration and surgical approaches. Eur Spine J.

[25] Choi KC, Kim JS, Ryu KS, Kang BU, Ahn Y, Lee SH (2013). Percutaneous endoscopic lumbar discectomy for L5-S1 disc herniation: transforaminal versus interlaminar approach. Pain Physician.

[26] John J (1984). Grading of muscle power: comparison of MRC and analogue scales by physiotherapists. Medical Research Council. Int J Rehabil Res.

[27] Toyone T, Tanaka T, Kato D, Kaneyama R, Otsuka M (2005). Patients' expectations and satisfaction in lumbar spine surgery. Spine (Phila Pa 1976).

[28] Krishnan A, Chauhan V, Degulmadi D (2023). Prodrome to seizure in transforaminal endoscopic surgery: a series of 9 cases. J Minim Invasive Spine Surg Tech.

[29] Krishnan A, Marathe N, Degulmadi D (2022). End-points of decompression of in lumbar transforaminal endoscopic spine surgery: a narrative review of objective and subjective criteria to prevent failures. J Minim Invasive Spine Surg Tech.

[30] Ostelo RW, Deyo RA, Stratford P (2008). Interpreting change scores for pain and functional status in low back pain: towards international consensus regarding minimal important change. Spine (Phila Pa 1976).

[31] Choi KC, Park CK (2016). Percutaneous endoscopic lumbar discectomy for L5-S1 disc herniation: consideration of the relation between the iliac crest and L5-S1 disc. Pain Physician.

[32] Song QC, Zhao Y, Li D (2021). Percutaneous endoscopic transforaminal discectomy for the treatment of L5-S1 lumbar disc herniation and the influence of iliac crest height on its clinical effects. Exp Ther Med.

[33] Vleeming A, Schuenke MD, Masi AT, Carreiro JE, Danneels L, Willard FH (2012). The sacroiliac joint: an overview of its anatomy, function and potential clinical implications. J Anat.

[34] Krishnan A, Kulkarni M, Singh M (2019). Trans-foraminal endoscopic uniportal decompression in degenerative lumbar spondylolisthesis: a technical and case report. Egyptian J Neurosurg.

[35] Ahn Y, Kim CH, Lee JH, Lee SH, Kim JS (2013). Radiation exposure to the surgeon during percutaneous endoscopic lumbar discectomy: a prospective study. Spine (Phila Pa 1976).

[36] Sousa JM, Serrano A, Nave A, Mascarenhas V, Nogueira P, Gamelas J, Guimarães Consciência J (2023). Transforaminal endoscopic approach to L5S1: imaging characterization of the lower lumbar spine and pelvis for surgical planning. World Neurosurg.

[37] Hsu HT, Chang SJ, Yang SS, Chai CL (2013). Learning curve of full-endoscopic lumbar discectomy. Eur Spine J.

[38] Wu XB, Fan GX, Gu X (2016). Learning curves of percutaneous endoscopic lumbar discectomy in transforaminal approach at the L4/5 and L5/S1 levels: a comparative study. J Zhejiang Univ Sci B.

[39] Yang JS, Liu KX, Kadimcherla P (2020). Can the novel lumboIliac triangle technique based on biplane oblique fluoroscopy facilitate transforaminal percutaneous endoscopic lumbar discectomy for patients with L5-S1 disc herniation combined with high iliac crest? Case-control study of 100 patients. Pain Physician.

[40] Fan G, Wang T, Hu S, Guan X, Gu X, He S (2017). Isocentric navigation of percutaneous endoscopic transforaminal discectomy at the L5/S1 level in difficult puncture cases: a technical note. Pain Physician.

[41] Chen Z, Wang X, Cui X, Zhang G, Xu J, Lian X (2022). Transforaminal versus interlaminar approach of full-endoscopic lumbar discectomy under local anesthesia for L5/S1 disc herniation: a randomized controlled trial. Pain Physician.

[42] Nie H, Zeng J, Song Y (2016). Percutaneous endoscopic lumbar discectomy for L5-S1 disc herniation via an interlaminar approach versus a transforaminal approach: a prospective randomized controlled study with 2-year follow up. Spine (Phila Pa 1976).

[43] Assis R, Nascimento W, Toulias A (2024). Transiliac endoscopic access for transforaminal approach at L5-S1: cases report. Coluna.

[44] Abbasi H (2020). Physiologic decompression of lumbar spinal stenosis through anatomic restoration using trans-Kambin oblique lateral posterior lumbar interbody fusion (OLLIF): a retrospective analysis. Cureus.

[45] Morimoto M, Tamaki S, Ogawa T (2023). Advantages of full-endoscopic trans-Kambin's triangle lumbar interbody fusion for degenerative spondylolisthesis: illustrative cases. NMC Case Rep J.

[46] Sousa JM, Silva JL, Gamelas J, Guimarães Consciência J (2023). Transiliac endoscopic-assisted L5S1 intraforaminal lumbar interbody fusion: technical considerations and potential complications. World Neurosurg.

[47] Naftalovich R, Singal A, Iskander AJ (2022). Enhanced recovery after surgery (ERAS) protocols for spine surgery - review of literature. Anaesthesiol Intensive Ther.

